# Gametocytes of the Malaria Parasite *Plasmodium falciparum* Interact With and Stimulate Bone Marrow Mesenchymal Cells to Secrete Angiogenetic Factors

**DOI:** 10.3389/fcimb.2018.00050

**Published:** 2018-03-01

**Authors:** Valeria Messina, Mauro Valtieri, Mercedes Rubio, Mario Falchi, Francesca Mancini, Alfredo Mayor, Pietro Alano, Francesco Silvestrini

**Affiliations:** ^1^Dipartimento di Malattie Infettive, Istituto Superiore di Sanità, Rome, Italy; ^2^Dipartimento di Oncologia e Medicina Molecolare, Istituto Superiore di Sanità, Rome, Italy; ^3^ISGlobal, Barcelona Ctr. Int. Health Res, Hospital Clínic - Universitat de Barcelona, Barcelona, Spain; ^4^AIDS National Center, Istituto Superiore di Sanità, Rome, Italy; ^5^Dipartimento di Biotecnologie Cellulari ed Ematologia, Umberto I - Policlinico di Roma, Rome, Italy; ^6^Centro de Investigação em Saúde da Manhiça, Maputo, Mozambique

**Keywords:** malaria, gametocytes, bone marrow, host-pathogen interactions, angiogenesis

## Abstract

The gametocytes of *Plasmodium falciparum*, responsible for the transmission of this malaria parasite from humans to mosquitoes, accumulate and mature preferentially in the human bone marrow. In the 10 day long sexual development of *P. falciparum*, the immature gametocytes reach and localize in the extravascular compartment of this organ, in contact with several bone marrow stroma cell types, prior to traversing the endothelial lining and re-entering in circulation at maturity. To investigate the host parasite interplay underlying this still obscure process, we developed an *in vitro* tridimensional co-culture system in a Matrigel scaffold with *P. falciparum* gametocytes and self-assembling spheroids of human bone marrow mesenchymal cells (hBM-MSCs). Here we show that this co-culture system sustains the full maturation of the gametocytes and that the immature, but not the mature, gametocytes adhere to hBM-MSCs via trypsin-sensitive parasite ligands exposed on the erythrocyte surface. Analysis of a time course of gametocytogenesis in the co-culture system revealed that gametocyte maturation is accompanied by the parasite induced stimulation of hBM-MSCs to secrete a panel of 14 cytokines and growth factors, 13 of which have been described to play a role in angiogenesis. Functional *in vitro* assays on human bone marrow endothelial cells showed that supernatants from the gametocyte mesenchymal cell co-culture system enhance ability of endothelial cells to form vascular tubes. These results altogether suggest that the interplay between immature gametocytes and hBM-MSCs may induce functional and structural alterations in the endothelial lining of the human bone marrow hosting the *P. falciparum* transmission stages.

## Introduction

The parasite *Plasmodium falciparum* causes the most severe form of malaria with around 438,000 deaths annually, mostly young children and pregnant women in sub-Saharan Africa (WHO, 2016). The global fight to control and to eventually eradicate malaria requires a multifaceted approach in which interventions that prevent transmission of Plasmodium from the infected individual to the mosquito have been prioritized. To this aim understanding the fundamental mechanisms of gametocyte maturation in the human host is essential to identify mechanisms that can be targeted by novel vaccines and drugs with transmission-blocking activity (Wells et al., 2009; Alonso et al., 2011; Lindblade et al., 2013).

*P. falciparum* has a complex life cycle, in which asexual replication and sexual development take place in red blood cells (RBCs) of the human host and sexual reproduction in the mosquito vector. While the asexual stages are responsible for malaria pathogenesis and the consequent morbidity and mortality, successful parasite transmission from humans to mosquitoes is dependent on the parasite sexual stages, termed gametocytes. Gametocytes undergo a development process classically divided into 5 morphological stages (I-V) that lasts about 10 days (Hawking et al., 1971), in which immature stages sequester in internal organs and only the mature stage V are released back into the blood stream where they can be harvested by the mosquito vector with the blood meal.

The presence of immature *P. falciparum* gametocytes in the bone marrow and spleen of infected individuals (Smalley et al., 1981; Farfour et al., 2012), has been recently confirmed by examination of autopsy specimens of different organs (Joice et al., 2014) and of bone marrow aspirates in children with nonfatal malarial anemia (Aguilar et al., 2014), independently demonstrating gametocyte enrichment in the bone marrow parenchyma. Morphology and stage specific staining in histological sections from some of these studies suggested that immature *P. falciparum* gametocytes undergo part of their development in the extravascular spaces of the host bone marrow (Farfour et al., 2012; Joice et al., 2014).

In the bone marrow parenchyma, specialized microenvironments, called niches, regulate hematopoietic stem cell (HSC) maintenance and function through an active crosstalk. Sacchetti et al. (2007) have shown that human CD45-146+/45– osteoprogenitor cells, also known as bone marrow mesenchymal stromal cells (hBM-MSCs), are able to transfer hematopoietic activity to an ectopic site *in vivo*, which awards them the status of stem cells. In the attempt to recapitulate the complexity of this niche, three-dimensional (3D) cultures of hBM-MSCs have been shown to reproduce cell behaviors more similar to what observed *in vivo* compared to bi-dimensional (2D) cultures (Baraniak and McDevitt, 2012; Laschke and Menger, 2017). 3D cultures for instance show an increased regenerative capacity through the secretion of anti-inflammatory, proangiogenic cytokines, and chemotactic factors (Baraniak and McDevitt, 2012). Several materials such as porous scaffolds and polymers, hydrogels, and ultra-low attachment cell culture plates are currently available to support *in vitro* 3D aggregates of MSCs with good dimensional control and tissue-like phenotypes (Benton et al., 2014; Sart et al., 2014). These methods take advantage of the natural self-assembly tendency typical of most cell types. Importantly, in these systems cells grow as spheroids and are able to generate their extracellular matrix and to communicate with each other as in their native environment (Sart et al., 2014). In the last decade, development of 3D cellular microenvironments with basement membrane extracts, termed BME/Matrigel, has progressed remarkably (Benton et al., 2014) and can be suitably tailored to reproduce tissue-like structures *in vitro*.

In addition to stromal cells, among the major players in the bone marrow growth and maintenance, there are endothelial cells, which are required for the development of a complex mature vascular system. hBM-MSCs directly interact, as pericytes, with endothelial cells, supporting the formation of the complex network of vessels, and facilitate hematopoietic progenitor cell maintenance in *in vitro* co-culture systems through the secretion of soluble factors (Wagner et al., 2007).

The emerging role of the bone marrow in hosting malaria parasites and providing a suitable environment for the maturation of gametocytes is attracting attention on the underlying molecular and physical cross talks between infected red blood cells and this tissue. In addition, the evidence of the localization of immature gametocytes in the bone marrow extravascular compartment raises questions on how parasites reach this site from the blood circulation and how they return to circulation at maturity. This dynamic behavior obviously suggests that an interplay with the endothelial barrier can be activated by the parasite at different stages of development from both the luminal and from the extravascular site. Evidence supporting the existence of such an interplay recently came from the report that vesicles secreted by asexual parasite-infected erythrocytes are able to increase permeability of the endothelial lining (Mantel et al., 2016).

To experimentally address these questions, we investigated the cellular interactions between human bone marrow endothelial and stromal cells and immature and mature *P. falciparum* gametocytes *in vitro*. These studies revealed that immature gametocyte-infected erythrocytes are able to cytoadhere to hBM-MSCs through trypsin-sensitive ligands on the infected erythrocyte surface. We developed a Matrigel™ 3D co-culture system mimicking features of the bone marrow stromal environment, wherein hBM-MSCs aggregate in clusters and the interacting gametocytes reach full maturation. We observed that hBM-MSCs are induced by gametocytes to increase production and secretion of a subset of cytokines and growth factors, some of which involved in angiogenesis, and that supernatants of the gametocyte-hBM-MSC co-cultures are functionally able to induce BM endothelial cells to promote formation of vessel like structures.

## Materials and methods

### Parasite culture and sexual stage induction

*P. falciparum* clones 3D7A (Walliker et al., 1987) and ItG (Ockenhouse et al., 1991) were cultured as previously described (Trager and Jensen, 1976). For parasite synchronization, cultures at 8–10% parasitaemia at 10% haematocrit were centrifuged at 800 rcf for 10 min through a 60% Percoll cushion and slow sedimenting schizonts used to reinvade fresh red blood cells. Induction of gametocytogenesis was performed at high parasitemia, as previously described (Lucantoni et al., 2015). Residual asexual stage parasites were cleared by incubation with N-Acetylglucosamine for 48 h to obtain enriched gametocyte cultures, which were maintained with daily change of medium until they reached the required maturation stages.

### Cell cultures and static adhesive assay

hBM-MSCs were isolated from healthy human donors according to Sorrentino et al (Sorrentino et al., 2008) and cultured in α-medium (Invitrogen, Carlsbad, CA, USA), 20% fetal calf serum (StemCell) in T75 flasks at 37°C in 5% CO_2_ atmosphere. Samples were obtained with informed written consent per institutionally approved protocols. Static cell binding assays were carried out using a modified version of a previously described method (Crabb et al., 1997). Briefly, 20,000 host cells were grown to confluence in a 24 well plate and exposed to TNF-α (0.5 ng/ml) for 12 h to increase expression of adhesive molecules or left untreated. Equal numbers (1 × 10^6^) of late trophozoites from synchronous asexual cultures of ItG, immature and mature 3D7 gametocytes were adjusted to 1% hematocrit and incubated for 2 h at 37°C in 5%CO_2_ atmosphere. After 2 washes with RPMI (30 min/wash) to remove unbound uninfected and infected erythrocytes, cell monolayers were fixed and Giemsa stained, and the numbers of bound parasites per mm^2^ of cell layer were counted. The trypsin cleavage assay was performed as described in Waterkeyn et al. (2000). Immortalized HBMEC-60 were kindly provided by Dr. E van der Schoot (CLB, Sanquin Blood Supply Foundation, The Netherlands). HBMEC-60 cells were routinely grown in 1% gelatin-coated culture flasks using endothelial cell growth medium (EBM-2 BulletKit, Lonza).

### Inhibition of parasite binding to hBM-MSCs

hBM-MSCs, grown to confluence in 96 well plate in α-medium (Invitrogen, Carlsbad, CA, USA) with 20% fetal calf serum (StemCell), were incubated for 60 min at 37°C in 5% CO_2_ atmosphere with the following anti-cadherin and anti-integrin antibodies: CD54, CD90, CD44, CD10, CD105, CD29, CD106, CD140a, CD140b, CD146, CD13, CD147, CD166, GD2, HLA-ABC, ICAM1, CD29, CD49-a,-c,-d, and CD54 (0.1 mg/1 × 10^6^ cells; BD, Pharmingen). After PBS washes, equal numbers (3 × 10^5^) of purified 3D7 stage II gametocytes and of ItG synchronous late trophozoites were incubated 2 h with hBM-MSCs at 37°C in 5% CO_2_ atmosphere. After removal of unbound parasites, cell monolayers were fixed and Giemsa stained, and the numbers of bound parasites per mm^2^ of cell layer were counted.

### 3D co-culture scaffold preparation

The 3D scaffolds were made into transwell cell culture inserts (8 μm pore size, Corning), using 50 μl of Corning Matrigel™ Matrix (10 mg/ml, Corning) according to standard procedures. Once gelled, 3000 hBM-MSCs were added to each transwell and incubated at 37°C in 5% CO_2_ atmosphere. After 48 h the hBM-MSCs medium inside the insert was replaced with 200 μl parasite complete medium containing 500,000 stage II gametocytes, while 1 ml of hBM-MSCs medium was added into the bottom of the plate wells. Every 48 h, cells from Matrigel™ Matrix were recovered using Corning Cell Recovery Solution (Corning), to monitor cell growth and viability by Giemsa staining.

### Cytokine profiling in MSCs culture supernatants

Supernatants from hBM-MSC cultures and hBM-MSCs/gametocyte co-cultures were collected at four different time points, every 48 h, and protein concentration was quantified using Quant-iT™ protein assay kit (Qubit; Molecular Probes, Invitrogen). Measurements of cytokine concentrations in culture samples were done simultaneously by bead-based sandwich immunoassays (Human Cytokine Magnetic 30-Plex Panel, Life Technologies). Data for each kit were analyzed as recommended by the manufacturers, with minor modifications: concentration of each analyte was obtained by interpolating fluorescence intensity to a 16-point dilution 1/2 standard curve supplied by the kit and calculated with the XPonent 3.1 Software. Data were normalized on the total number of cells. Outliers and samples dilutions with MFI (Fluorescence) equal or higher to mean+2 SD (standard deviation) of the blanks, were discarded.

### Immunofluorescence analysis

The morphology of hBM-MSCs and gametocytes in 3D matrigel scaffolds were visualized under confocal microscope. Corning Matrigel Matrix (10 mg/ml, Corning) was seeded into the chamber slide wells. After polymerization, hBM-MSCs and stage II gametocytes were seeded together. After 48 h, scaffolds were fixed, permeabilized and incubated with primary antibodies: Texas Red-X Phalloidin 1:100 (TermoFisher scientific), anti-CD90 1:100 (Abcam) and anti-Pfg27 1:200 (Olivieri et al., 2009). Images were taken at 20 × magnification on an inverted microscope (Olympus) equipped with a confocal spectral imaging system (Olympus Fluoview 1000, Tokyo, Japan) using a (Olympus) planapo objective 60 × oil A.N. 1,42. Excitation light was obtained by an Argon Ion Laser (488 nm) for Alexa Fluor 488 and a Diode Laser HeNe (561 nm) for Alexa Fluor 555. Alexa Fluor 488 emission was recorded from 500 to 567 nm; Alexa Fluor 555 red emission was recorded from 583 to 628 nm. Recorded images have an optical thickness of 0.42 μm.

### Tube formation assay

hBM-MSCs were seeded at 20,000 cells/well onto the surface of a 24 well plate. After reaching confluence, 500,000 stage II gametocytes were added to the well and co-cultured with hBM-MSCs for 48 h in α -medium (Invitrogen, Carlsbad, CA, USA) with 20% fetal calf serum (StemCell) at 37°C in 5% CO_2_ atmosphere. The derived conditioned medium was collected and used for the tube formation assay. HBMEC-60 were seeded at 20,000 cells/well onto the surface of polymerized Corning Matrigel Matrix (10 mg/ml, Corning), inside a transwell support (0.4 μm pore size, Corning), using endothelial cell growth medium (EBM-2 BulletKit, Lonza). Conditioned media were added to a multiple well plate well, subsequently adding the transwell insert with polymerized Matrigel. Endothelial cells were seeded into the transwell insert, adding EBM-2 medium. The plate was incubated for 12 h to allow formation of capillary-like structures. Phase-contrast images were captured by using an inverted light microscope (Evos, Invitrogen) at 4 × magnification. Images were analyzed and quantified by using the image J analysis software (angiogenesis analyzer script) (Guidolin et al., 2004). The readouts of the image analysis software include tubule and net characteristics. Data were shown as means ± standard errors.

### Statistical methods

Data are expressed as mean ± standard deviation (SD). Analysis of statistical significance was performed by Student's *t*-test followed by Tukey test. Differences in cytokines secretion between the two groups (cocultures of hBM-MSC in presence/absence of gametocytes) were analyzed using the non-parametric Mann-Whitney test. Statistical significance was defined as *p* < 0.05. Prism® 5 software (GraphPad Software, San Diego, CA, USA) was used for statistical analysis.

## Results

### *P. falciparum* immature gametocytes adhere to hBM-MSCs through trypsin sensitive ligands

The ability of immature gametocytes to cytoadhere like asexual stage parasites to human endothelial cells has been the object of several studies. Apart from one report (Rogers et al., 2000), all subsequent studies failed to show binding of red blood cells infected by immature gametocytes, produced by laboratory strains and by one wild isolate, to human endothelial cell lines derived from bone marrow, and other organs (Rogers et al., 1996; Silvestrini et al., 2012; Tibúrcio et al., 2013). The recent demonstration of immature gametocyte enrichment in the bone marrow stroma however prompted us to investigate whether this phenomenon could be mediated by direct cellular interaction(s) between hBM-MSCs and immature gametocytes. To directly test this hypothesis, static adhesion assays were used to compare cytoadhesion of asexual stages and immature and mature gametocytes on monolayers of hBM-MSCs and human bone marrow derived endothelial cells (HBMEC-60) (Rood et al., 2000). The hBM-MSCs used in this study, obtained from healthy human bone marrow aspirates as previously described (Sorrentino et al., 2008), were routinely characterized for the expression of stromal markers (e.g., CD105^+^, CD90^+^, CD45^−^) (Supplementary Figure 1), for the ability to maintain their tri-lineage differentiation potential in osteocytes, chondrocytes and adipocytes and to support hematopoietic colonies (Sorrentino et al., 2008; Signore et al., 2012).

**Figure 1:**
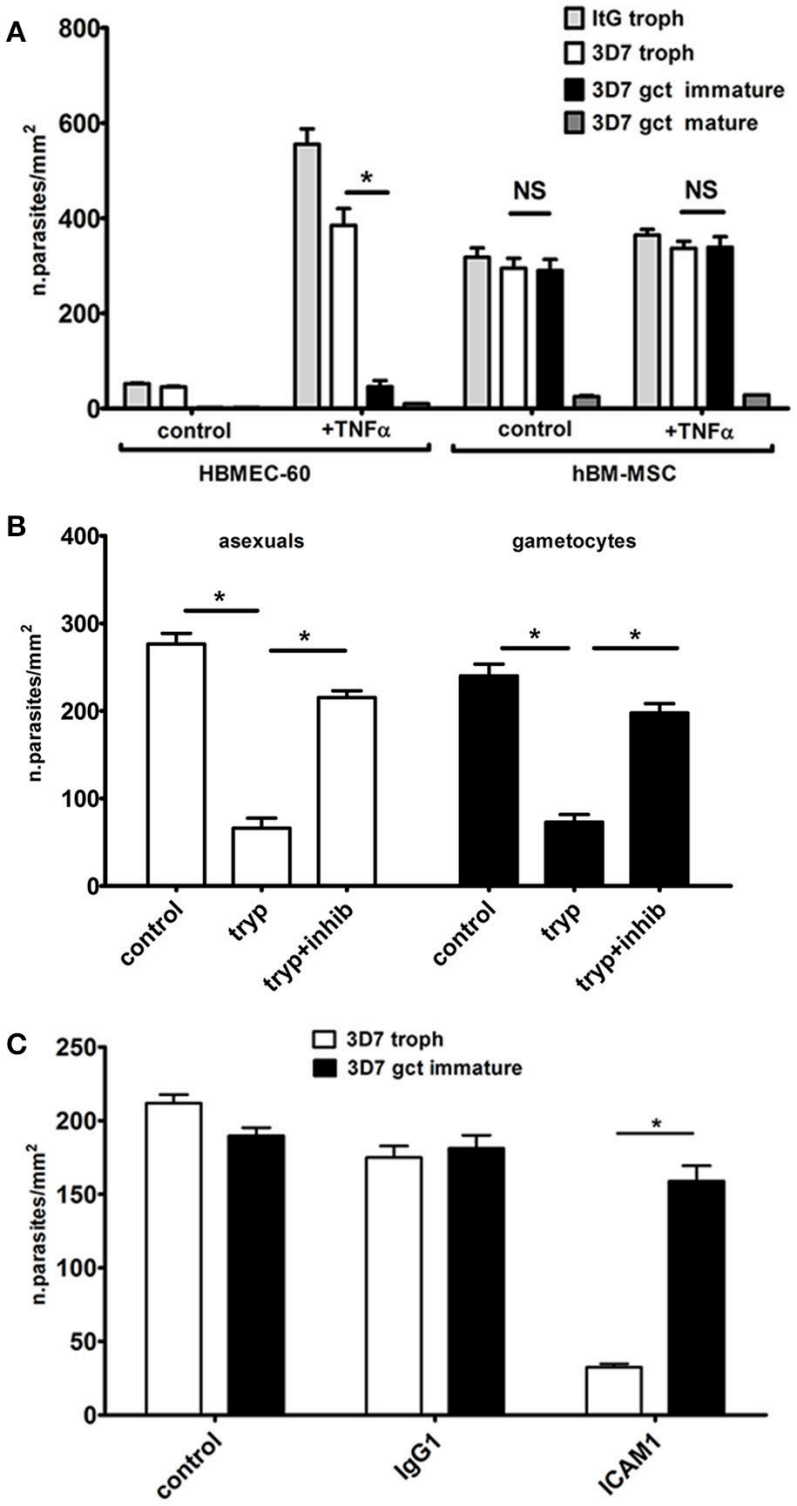
Static adhesion assays. **(A)** Cytoadhesion of erythrocytes infected by *P. falciparum* asexual and sexual stages on hBM-MSC and HBMEC-60 monolayers (*N* = 4; *n* = 3). The asterisks represent significant differences (^*^p < 0.05). **(B)** Cytoadhesion of erythrocytes infected by immature gametocytes and asexual trophozoites after trypsin surface treatment (*N* = 3; *n* = 3). **(C)** Cytoadhesion of erithrocytes infected by trophozoites and immature gametocytes incubated with anti-ICAM1 and control antibodies (*N* = 3; *n* = 3).

In our cytoadhesion experiments the *P. falciparum* clone ItG, a reference clone for asexual stage parasite adhesion (Ockenhouse et al., 1991), was used besides the gametocyte producer clone 3D7. Results (Figure 1A) clearly showed that stage V gametocytes failed to bind to the HBMEC-60 or hBM-MSC monolayers, confirming previous observations conducted on several endothelial host cell lines (Silvestrini et al., 2012). In contrast, immature gametocytes showed a high level of adhesion on the hBM-MSC monolayers, comparable to that of asexual stages, while a significantly lower adhesion on the HBMEC-60 monolayers was confirmed (Silvestrini et al., 2012). Moreover, adhesion of the immature gametocytes was not affected by TNF-α stimulation, necessary for asexual binding (Figure 1A).

To investigate the nature of the adhesive phenotype on hBM-MSCs, intact, live immature gametocyte-infected erythrocytes were subjected to a trypsin shaving treatment before the cell binding assay. A threefold drop in the binding efficiency of the immature gametocytes was measured after trypsin treatment compared to the untreated control and to treatment with trypsin plus soybean trypsin inhibitor (Figure 1B). This experiment provides evidence that adhesion is mediated by trypsin-sensitive proteins exposed on the surface of the gametocyte-infected erythrocyte, an observation that intriguingly contrasts with the lack of described gametocyte adhesins (Dinko et al., 2016). The same experiment was conducted in parallel with erythrocytes infected with asexual stage trophozoites. Binding efficiency of these parasites was comparable to that of immature gametocytes (Figure 1A) and it was also significantly reduced by surface trypsin treatment (Figure 1B).

As this observation suggested the involvement of the main parasite ligand used for asexual stage cytoadhesion, PfEMP-1 (Rowe et al., 2009), the possibility that residual PfEMP1 molecules on the surface of immature gametocyte infected red blood cell were responsible for the observed binding to hBM-MSCs was tested by incubating asexual and sexual stage parasites with antibodies against ICAM1, a major PfEMP1 human ligand. The experiment clearly showed that the anti-ICAM1 antibodies, but not the Ig G1 isotype control, almost abolished adhesion of the asexual stage parasites but did not affect the binding of the immature gametocytes (Figure 1C). This result suggests the existence of a different host-parasite ligand interaction responsible for gametocyte binding to hBM-MSCs.

To identify possible hBM-MSC receptors involved in immature gametocyte adhesion we tested the inhibitory effect of 19 antibodies specific for cadherins and integrins expressed on the hBM-MSC surface (Signore et al., 2012). Failure of any of these antibodies to inhibit immature gametocyte binding to hBM-MSCs however suggested that these ligands do not play a major, if any, role as human receptors for immature gametocyte adhesion (Supplementary Figure 2).

### Gametocytes undergo full maturation in a 3D co-culture system with human bone marrow mesenchymal cells

**Figure 2:**
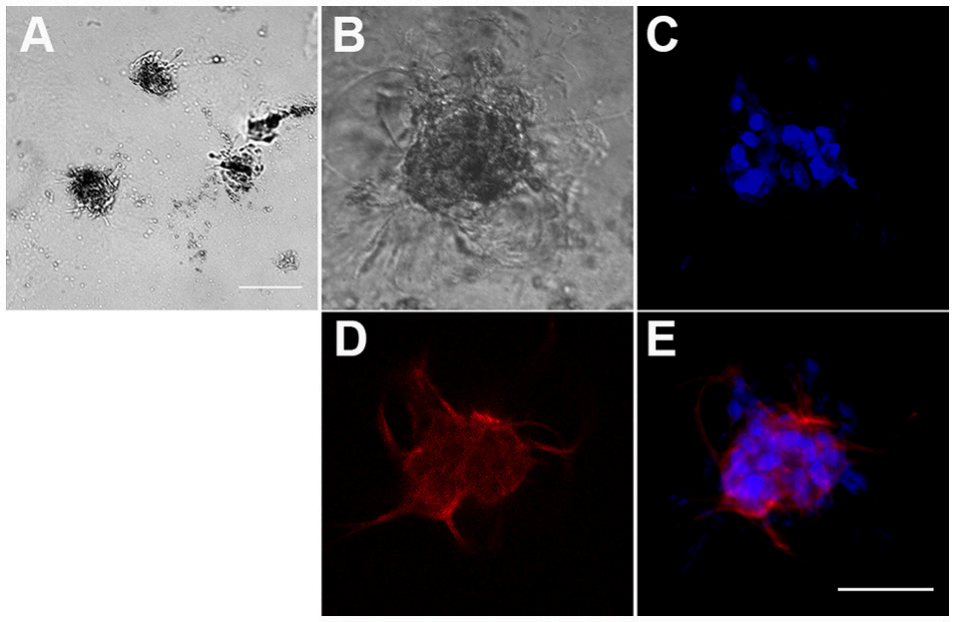
Formation of three-dimensional hBM-MSC spheroids in Matrigel scaffold**. (A)** Phase contrast microscopy of hBM-MSC spheroids in Matrigel. Scale bar: 1,000 μm. **(B–E)** A representative hBM-MSC spheroid in phase contrast **(B)**, with the nuclear stain Hoechst blue **(C)**, stained with an anti-CD90 specific antibody red **(D)**, and merge **(E)**. Scale bar: 100 μm.

To characterize the interaction between hBM-MSCs and *P. falciparum* gametocytes in a more physiological context than the above cell monolayers, we first developed a 3D culture system, more suitable to study complex cell–cell and cell–matrix interactions, exploiting the ability of hBM-MSCs to self-assembly in spheroids and to communicate with each other as in their natural environment (Sart et al., 2014). Morphological analysis of hBM-MSC cultures in Matrigel™ Basement Membrane Matrix revealed that in this condition cells were indeed able to self-aggregate into clusters of different size after 2 days (Figure 2). Seeding a mean number of 3,000 cells/100 μl in the upper chamber of the Transwell insert of a 24 well plate, we routinely obtained an average of 109.6 ± 33.6 spheroids (Figure 2A), with an average diameter of 211.27 ± 61.9 μm, as measured by bright field microscopy (Figure 2B).

**Figure 3:**
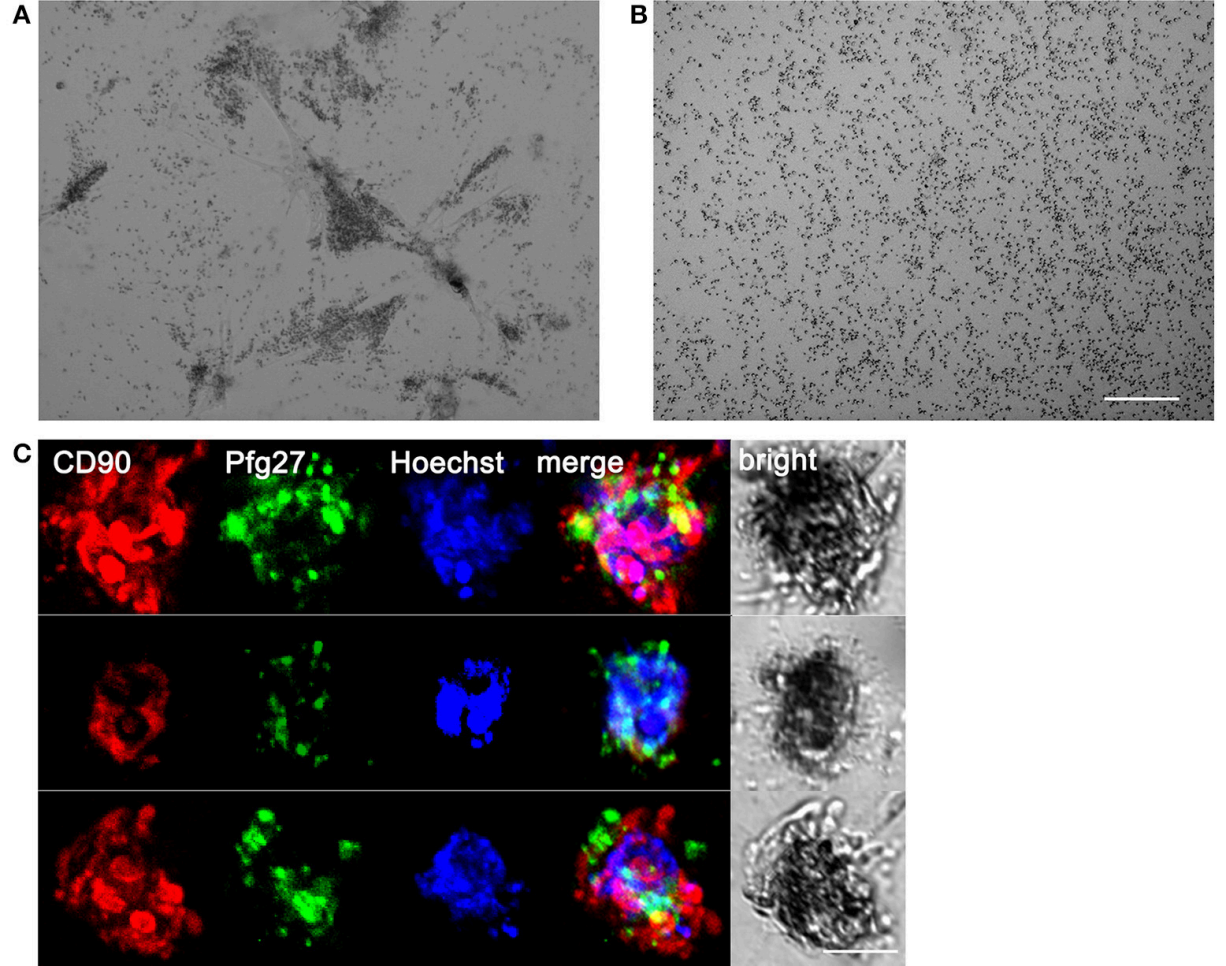
Association of gametocytes with hBM-MSC spheroids in the 3D co-culture system. **(A)** Phase contrast microscopy showing stage II gametocytes associated with hBM-MSC spheroids in Matrigel scaffold compared with **(B)** gametocytes randomly distributed in Matrigel scaffold without cells. Scale bar: 200 μm. **(C)** Three representative co-culture spheroids stained with anti-CD90 antibodies (red) to detect mesenchymal cells, anti-Pfg27 antibodies (green) to detect gametocytes and Hoechst (blue) to stain nuclei. Scale bar: 50 μm.

The system was used to establish a 3D co-culture system of hBM-MSCs and *P. falciparum* gametocytes. To this aim, purified stage II gametocytes were seeded with hBM-MSCs in Matrigel™ Basement Membrane Matrix in the upper chamber, constituted by a 8 μm Transwell insert, in a well of a 24 well plate (Supplementary Figure 3). After 48 h the hBM-MSCs produced the clusters described above. Microscopy inspection of the co-cultures from 48 h onwards revealed that the immature gametocytes were found to accumulate in close contact with the hBM-MSC spheroids (Figure 3A). This was confirmed by immunofluorescence analysis using antibodies against the stromal marker CD90 and the gametocyte specific protein Pfg27 (Alano et al., 1991) (Figure 3B). In contrast, when the same number of gametocytes were seeded in the absence of the hBM-MSC cells, they were found evenly distributed in the matrix (Figure 3B), indicating that presence of the hBM-MSC spheroids was necessary for gametocyte aggregation and suggesting that this was mediated by the parasite host cell physical contacts described above.

**Figure 4:**
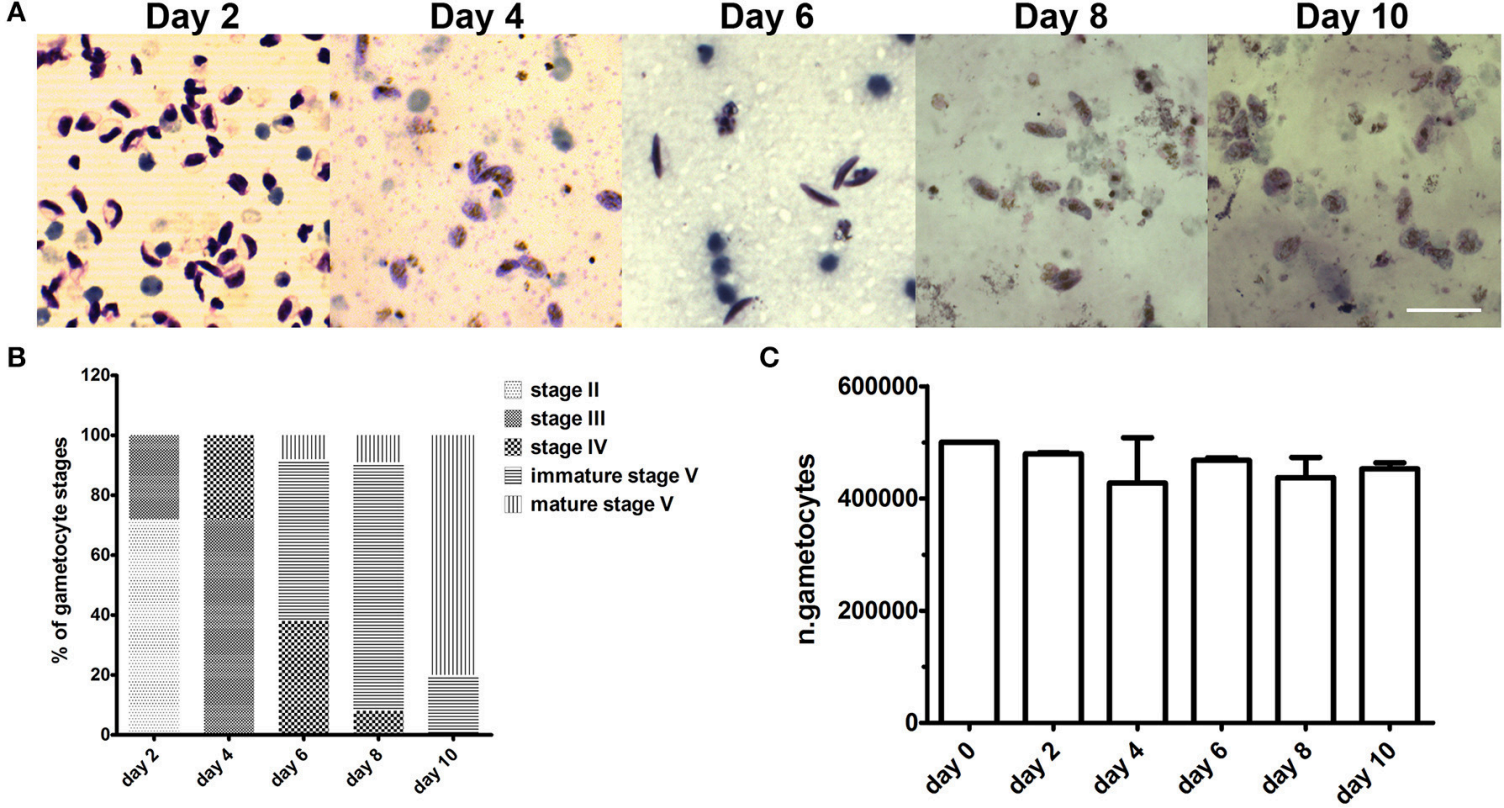
Gametocyte development in the 3D co-culture system. **(A)** Representative images of Giemsa-stained parasites recovered from the Matrigel scaffold after 2, 4, 6, 8, and 10 days of co-culture with hBM-MSCs. Scale bar: 20 μm. **(B)** percentage of different gametocyte stages as measured in Giemsa stained smears of parasite samples recovered from Matrigel after 2, 4, 6, 8, and 10 days of co-culture with hBM-MSCs (*N* = 3; *n* = 2). **(C)** Mean numbers of gametocytes counted in the above samples. Error bars are SD (*N* = 3; *n* = 2).

To evaluate if the 3D system was able to support gametocyte development until maturation, the co-culture was maintained for 10 days and examined morphologically every 48 h. Microscopy examination of parasites recovered from the hydrogel and stained by Giemsa showed that gametocytes were able to mature from stage II until stage V (Figures 4A–C). At day 10, the observation of spherical parasite forms suggested that gametocytes had reached maturity as these represented macrogametes formed by the spontaneous induction of mature female gametocytes. This was directly confirmed by inducing gametogenesis from parasites recovered at day 8 and 10 and observing that the sexual stages were functionally able to transform into gametes and, in the case of male gametocytes, to produce motile gametes in exflagellation centers. In this experiment, counts of gametocytes recovered from the Matrigel scaffold at the different time points showed that number of gametocytes did not decrease in the 10 day time course (Figure 3C), indicating that the co-culture system also quantitatively support gametocyte development.

In conclusion, these results establish a 3D co-culture system of *P. falciparum* gametocytes with hBM-MSCs in which sexual stages are physically associated to the host cell spheroids and in which stage progression and timing of gametocytogenesis are indistinguishable from those of gametocytes in conventional *in vitro* cultures. The fact that virtually no loss of gametocyte number is observed in the 10 day long maturation time indicate that the hydrogel-based hBM-MSCs cultures provide a suitable microenvironment supporting host-parasite interactions and full gametocyte maturation.

### Immature gametocytes enhance hBM-MSC secretion of pro-angiogenic factors

**Figure 5:**
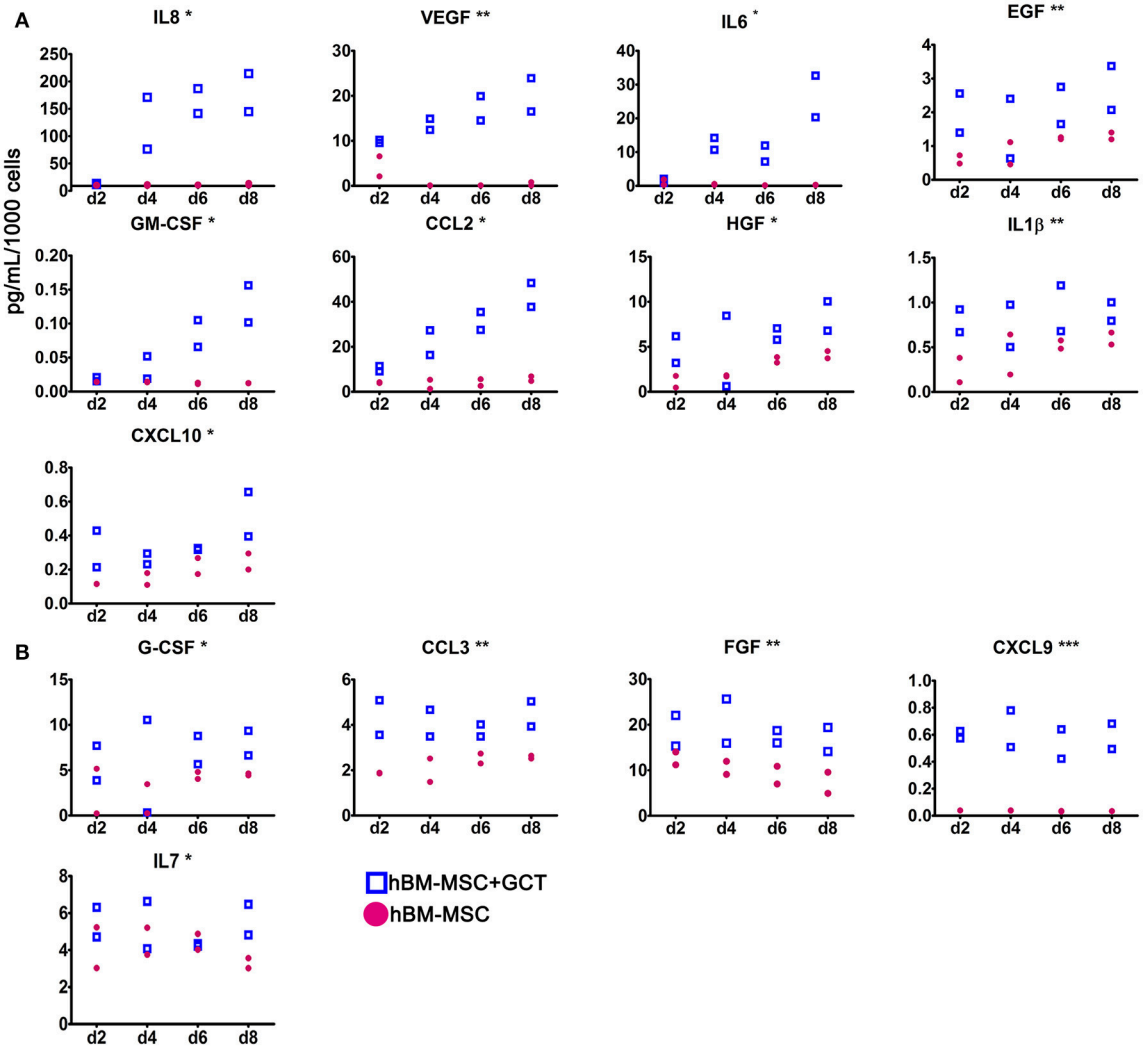
Secretion of cytokines and growth factors by hBM-MSCs in the co-culture system. Concentration of the indicated cytokines and growth factors secreted by hBM-MSCs alone (red dots) or co-cultivated with gametocytes (empty squares), collected at day 2, 4, 6, and 8 of culture. **(A)** Angiogenic factors showing a similar trend, increasing from day 2 to day 8, unlike that of FGF, CCL3, G-CSF, CXCL9 and IL7 **(B)**. Y axis indicates the mean of pg/ml/1,000 cells/48 h; error bar is SD (*N* = 2; *n* = 2), (^***^p < 0.001, ^**^p < 0.01, ^*^p < 0.05).

The 3D co-culture system was used to investigate the regulatory interplay relevant to gametocyte development in the BM microenvironment, specifically investigating whether immature gametocytes could affect the interplay between hBM-MSCs and endothelial cells. To this aim we measured the response of hBM-MSCs to the presence of immature gametocytes in the ability to secrete soluble cytokines, particularly those regulating angiogenesis. To measure levels of these soluble factors, we used a multiplex assay for 30 cytokines and growth factors, 20 of which have been described for being involved in angiogenesis (G-CSF, GM-CSF, IL-1RA, IL-1β, IL-2, IL-6, IL-8, IL-12, IL-15, IL-17, CCL11, CXCL9, CXCL10, CCL2, CCL3, CCL5, VEGF, EGF, FGF, and HGF (Angiolillo et al., 1997; Airoldi and Ribatti, 2011; Bouchentouf et al., 2011; Zhao et al., 2014; Yadav et al., 2015; Ridiandries et al., 2016; Huang et al., 2017). In this experiment, hBM-MSCs and stage II gametocytes or control uninfected erythrocytes were seeded in the Matrigel scaffold, layered on transwell chambers and co-cultured for 8 days. Supernatants of the co-culture wells were collected every 48 h and cytokine/growth factor concentrations determined in two biological replicates of the time course (Supplementary Table 1). In order to identify factors differentially produced in presence of developing gametocytes, we both evaluated the statistical significance in the global cytokines production in presence/absence of gametocytes and analyzed the production profile during the time course. The experiment showed that the concentration of 13 of the above 20 angiogenic factors was significantly higher (*p* < 0.05) in the supernatants of the hBM-MSCs co-cultured with immature gametocytes compared to those cultivated with uninfected red blood cells, thus indicating that hBM-MSC secretion of these factors was stimulated by the presence of gametocytes. In contrast, only 1 (IL7) of the remaining 10 factors analyzed exhibited a similar role (Table 1) Profile inspection further revealed that overproduction of 9 of the above 13 angiogenic factors showed a similar trend, steadily increasing from day 2 to day 8, in parallel to gametocyte maturation (Figure 5A), unlike that of FGF, CCL3, G-CSF, and CXCL9 and the non-angiogenetic IL7 (Figure 5B).

**Table 1:** Factors differentially secreted in coculture in presence or absence of developing gametocytes.

**Name**	**Class**	**Involved in angiogenesis**	**Significantly produced between cocultures**
CXCL9	Chemokine	Yes	^***^
CCL3	Chemokine	Yes	^**^
IL1B	Cytokine	Yes	^**^
VEGF	Growth Factor	Yes	^**^
EGF	Growth Factor	Yes	^**^
FGF	Growth Factor	Yes	^**^
CCL2	Chemokine	Yes	^*^
CXCL10	Chemokine	Yes	^*^
IL8	Cytokine	Yes	^*^
IL6	Cytokine	Yes	^*^
HGF	Growth Factor	Yes	^*^
GM-CSF	Growth Factor	Yes	^*^
G-CSF	Growth Factor	Yes	^*^
CCL5	Chemokine	Yes	ns
CCL11	Chemokine	Yes	ns
IL1RA	Cytokine	Yes	ns
IL2	Cytokine	Yes	ns
IL12	Cytokine	Yes	ns
IL15	Cytokine	Yes	ns
IL17	Cytokine	Yes	ns
IL7	Chemokine	No	^*^
IFNa	Cytokine	No	ns
IL2R	Cytokine	No	ns
IL4	Cytokine	No	ns
IL5	Cytokine	No	ns
CCL4	Cytokine	No	ns
IL10	Cytokine	No	ns
IL13	Cytokine	No	ns
IFNg	Cytokine	No	ns
TNFa	Cytokine	No	ns

*** *p < 0.001*,

** *p < 0.01*,

* *p < 0.05*.

### EC tube morphogenesis is induced by hBM-MSCs/immature gametocyte 3D co-culture supernatants

To functionally investigate whether the gametocyte-induced secretion of angiogenetic factors by hBM-MSCs affected endothelial cell physiology, we measured the influence of these factors on the ability of BM-derived endothelial cells to form capillary-like structures. The “tube formation assay,” routinely used to measure this parameter (Montesano et al., 1983), was conducted in transwell chambers with HBMEC-60 (Rood et al., 2000) exposed for 16–18 h to supernatants obtained from hBM-MSCs co-cultured with stage II-III gametocytes for 48 h, from day 2 to day 4, or with asexual stage parasites. Other controls were represented by exposing HBMEC-60 cells to MSC and EBM2 synthetic culture media.

**Figure 6:**
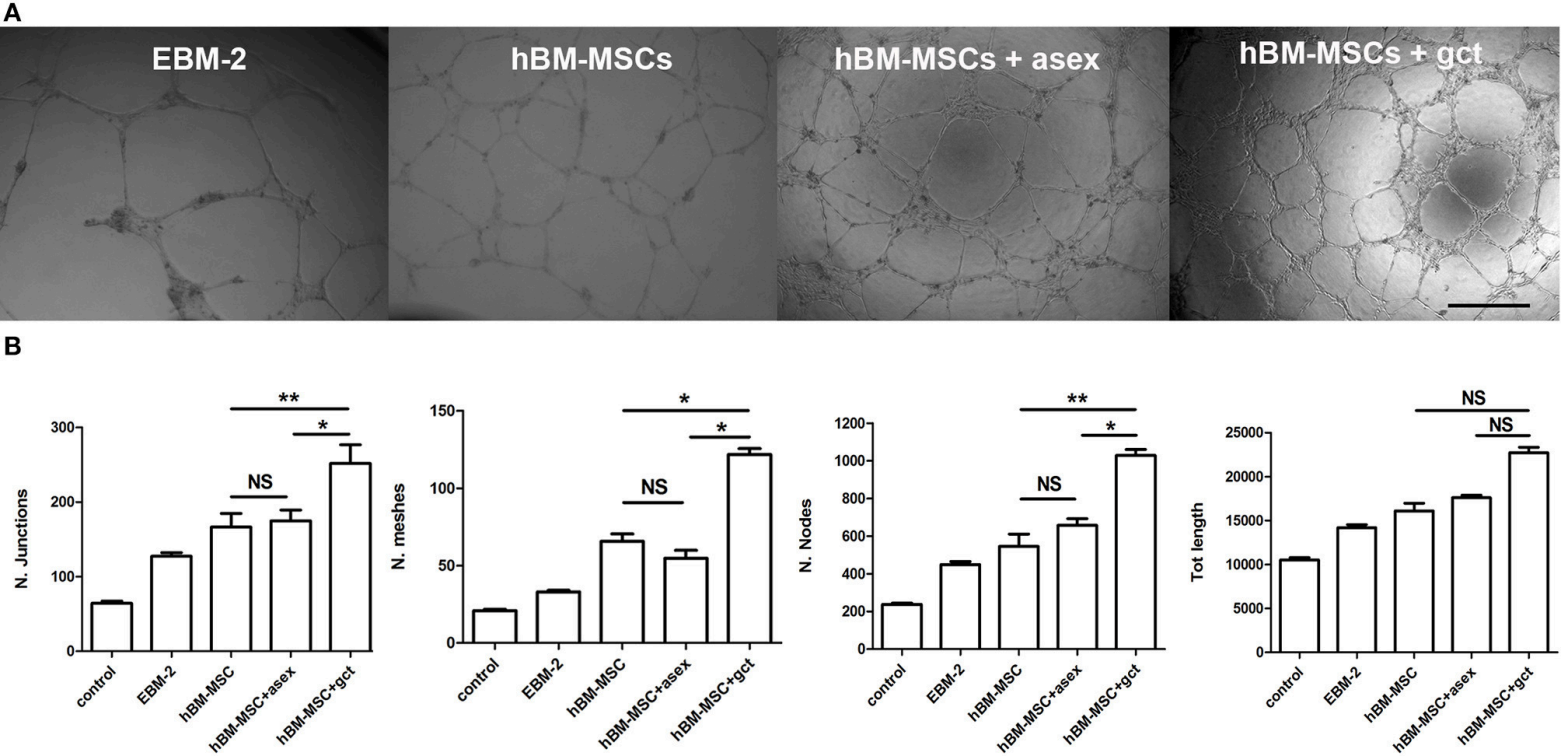
Tube formation assay with human bone marrow derived endothelial cell line (HBMEC-60). **(A)** Representative images of endothelial tube formation in Matrigel in presence of the indicated conditioned media. EBM-2, medium from culture of endothelial cells; hBM-MSC, medium from culture of hBM-MSCs; hBM-MSC + asex, medium from a co-culture of hBM-MSCs with asexual stage parasites; hBM-MSC + gct, medium from a co-culture of hBM-MSCs with immature gametocytes. Scale bar: 500 μm. **(B)** Plot of the four indicated parameters measured in the tube formation experiments on HBMEC-60 incubated with the above conditioned media. Data are represented as mean ± SD of the ratio on the control (*N* = 3; *n* = 3). The asterisks represent significant differences (^**^p < 0.01, ^*^p < 0.05).

The network of HBMEC-60 tube formation was analyzed measuring parameters such as number of nodes and junctions, meshes, and total tubule length (Supplementary Figure 4) between samples (Figure 6A). The result of this experiment was that the conditioned medium from hBM-MSCs in co-culture significantly enhanced the angiogenesis process by all parameters analyzed (*p* < 0.01, Figure 6B) and, noticeably, for 3 out of 4 parameters, this effect was significantly higher (*p* < 0.05) with the supernatant of hBM-MSCs co-cultured with immature gametocytes compared to that of hBM-MSCs co-cultivated with asexual stage parasites.

## Discussion

The recent observations that developing gametocytes of *P. falciparum* preferentially accumulate in the hBM (Smalley et al., 1981; Farfour et al., 2012; Aguilar et al., 2014; Joice et al., 2014), and particularly in the BM extravascular spaces (Farfour et al., 2012; Joice et al., 2014), open key questions and call for appropriate tools to elucidate the underlying molecular mechanisms. The hBM microenvironment contains a complex set of cellular, chemical, structural, and physical cues necessary to maintain the viability and function of the hematopoietic system (Nwajei and Konopleva, 2013). Our work addressed the host-parasite cross talk established by *P. falciparum* gametocytes in this unique microenvironment by developing an *in vitro* model to study host-parasite physical interactions and the modulation of host cytokine production between immature gametocytes and mesenchymal cells, the key components of the hBM parenchyma.

Development of our hBM-MSC/*P. falciparum* 3D co-culture system relied on the use of Matrigel™ as a physical scaffold. In the past, Matrigel™ has been used as substrate for development of axenic cultivation of *P. falciparum* asexual stages, in which stage II gametocytes were observed after 36–44 h of incubation (Trager and Williams, 1992). Although *P. falciparum* gametocyte maturation *in vitro* does not require presence of host cells other than erythrocytes, we show here that our co-culture model quantitatively and qualitatively supports the complete development of functionally mature stage V gametocytes. This represents, to our knowledge, the first *in vitro* model reproducing *P. falciparum* gametocytogenesis in presence of the major cellular component of the hBM parenchyma.

In this co-culture system, the majority of parasites were found in close contact with the hBM-MSC clusters most likely through the adhesive, trypsin sensitive interactions between *P. falciparum* gametocytes and hBM-MSCs revealed in cytoadhesion assays. This observation revises the current notion that immature gametocytes are unable to establish adhesive interactions with human cells. After a single report describing *in vitro* adhesion of *P. falciparum* immature gametocytes with endothelial and stromal hBM cells (Rogers et al., 2000), several studies failed to detect any adhesion of immature and mature sexual stages to endothelial cells of various human organs, including hBM (Silvestrini et al., 2012). This was consistent with the failure to detect parasite antigens exposed on the surface of immature gametocyte-infected erythrocytes (Saeed et al., 2008). The use for the first time in gametocyte cytoadhesion assays of primary hBM-MSCs enabled us to show here that immature, but not mature, gametocytes adhere *in vitro* to this human cell type with a similar efficiency than asexual stages, and that this binding is reversed by trypsin treatment of the parasitized RBCs.

Although immature gametocyte-infected erythrocytes have been shown to lack knobs, the main cell surface modifications mediating adhesion of asexual stage parasites to endothelial cells, and to minimally, if at all, expose the parasite adhesion ligand PfEMP1 (Tibúrcio et al., 2013), a hypothetical involvement in the observed binding to hBM-MSCs of residual PfEMP-1 surface molecules on immature gametocytes has been ruled out by the failure of anti-ICAM-1 antibodies to prevent this binding. On the other hand, the failure to interfere with gametocyte adhesion to hBM-MSCs by any of the 19 tested antibodies specific for major hBM-MSC antigens, suggests the implication of different ligand-receptor pair(s) in gametocyte binding. Nature of these ligands is still elusive, but possible candidates are suggested by the recently described interaction of the surface chemochine CX3CL1, which is exposed on hBM-MSCs (Honczarenko et al., 2006) and the gametocyte exported proteins PfGEXP07 and PfGEXP10 (Silvestrini et al., 2010; Hermand et al., 2016) and by ongoing work revealing surface antigens on immature gametocyte-infected red blood cells either by immune sera reactivity (M. Marti and T. Bousema, personal communication) or by mass spectrometry identification of surface trypsin-shaved proteins (E. Pasini and P. Alano, personal communication).

As hBM-MSCs represent a structural bone marrow population supporting hematopoiesis and immune-modulation through the secretion of a number of cytokines and growth factors (Spees et al., 2016), we investigated the profile of cytokine secretion by hBM-MSCs in co-culture with immature gametocytes. Aware of the fact that free haemozoin can dysregulate immune response (Pathak and Ghosh, 2016), we controlled that this component was virtually absent in our gametocyte samples, ruling out an effect of that confounding element in our study. This experiment revealed that immature gametocytes induced an increase in the release of 14 out the 30 factors tested. Although many of the secreted host factors may show pleiotropic effects, it was intriguing that 13 of them had been described to directly or indirectly contribute to regulate angiogenesis: CCL2, CCL3, CXCL9, CXCL10, IL-1β, IL-6, IL-8, VEGF, EGF, FGF, GM-CSF, G-CSF, and HGF (Gacche and Meshram, 2014). It is noticeable that among these factors, secretion of IL-8 (CXCL8), a potent promoter of angiogenesis by direct stimulation of hBM-MSC VEGF secretion (Hou et al., 2014), showed the highest fold increase (87.3 fold). In addition, the observation that the secretion profile of 8 of the 12 active angiogenetic factors closely paralleled the progression of gametocyte maturation in the co-culture system, with a peak on day 8, suggests that gametocytes exert during their maturation a progressively stronger induction of hBM-MSC secretion of those factors.

A role in angiogenesis of secreted factors upon gametocyte stimulation was functionally confirmed observing the increased vessel-like net organization produced by hBM endothelial cells exposed to hBM-MSC co-culture supernatants. This experiment also showed that factors promoting *in vitro* angiogenesis were more efficiently induced by gametocytes than by asexual stages.

The paucity of *in vivo* information on the parasite infected hBM microenvironment makes it difficult to firmly draw a parallel between our observations and specific events occurring in the *P. falciparum* infected hBM. Nevertheless, our work proposes that a parasite-host interplay in the hBM extravascular space may remodel the endothelial lining from the abluminal side, representing another mechanism used by the parasite to affect endothelium physiology, distinct from the increase in endothelial permeability induced by the circulation in the microvascular lumen of parasite borne membrane vesicle carrying specific microRNAs (Mantel et al., 2016).

The analysis of autopsy samples of *P. falciparum* infected bone marrow measured that gametocytes preferentially localize in proximity to the erythroblastic islands, the morphologically distinguishable niches for erythropoiesis, with some gametocytes developing inside immature erythroid precursors (Joice et al., 2014). It has been reported that erythroblastic islands are formed away from the sinusoids and progressively migrate toward the sinusoid lumen as erythroid maturation proceeds (Yokoyama et al., 2003; Manwani and Bieker, 2008). The observed localization of immature gametocytes and the gametocyte-induced increase of angiogenic factor secretion by hBM-MSCs may lead to speculate that a coordination between gametocyte development, island migration and cytokine/growth factor secretion is activated to promote the process of intravasation and re-entry in circulation of the stage V gametocytes. The ability of gametocytes to indirectly prime angiogenesis through the interaction with hBM-MSCs, as seen in tumor associated neo vascularization induced by the cancer cell-hBM-MSC interplay (Suzuki and Sun, 2011), may therefore be an additional, or alternative, mechanism to reorganize the vascularization of the stromal compartment to ensure a successful gametocyte maturation and release in the blood stream.

In conclusion, this study provides insights in the mechanisms of maintenance and development of the *P. falciparum* transmission stages in the human bone marrow. Our results, obtained from a novel *in vitro* 3D co-culture model, reveal for the first time that immature gametocytes physically interact with clusters of hBM-MSCs and prime the secretion of cytokines and growth factors. Ability of most of these factors to stimulate angiogenesis suggests that the immature gametocyte-mesenchymal cell interplay may facilitate release of the mature gametocytes from the bone marrow sequestration sites into the blood stream to ensure parasite transmission to the insect vector and perpetuation of the malaria parasite life cycle.

## Author contributions

FS, VM, MV, and PA designed the research. VM, MV, MR, MF, and FS performed the research. FM contributed new reagents and analytic tools. VM, MV, AM, FS, and PA analyzed the data. FS, VM, MV, and PA wrote the paper.

### Conflict of interest statement

The authors declare that the research was conducted in the absence of any commercial or financial relationships that could be construed as a potential conflict of interest.
