# Supplementary figures and images for: Gametocytes of the Malaria Parasite Plasmodium falciparum Interact With and Stimulate Bone Marrow Mesenchymal Cells to Secrete Angiogenetic Factors
Source: Front Cell Infect Microbiol. 2018 Mar 1;8:50. doi: 10.3389/fcimb.2018.00050 (PMC5838020; doi:10.3389/fcimb.2018.00050)

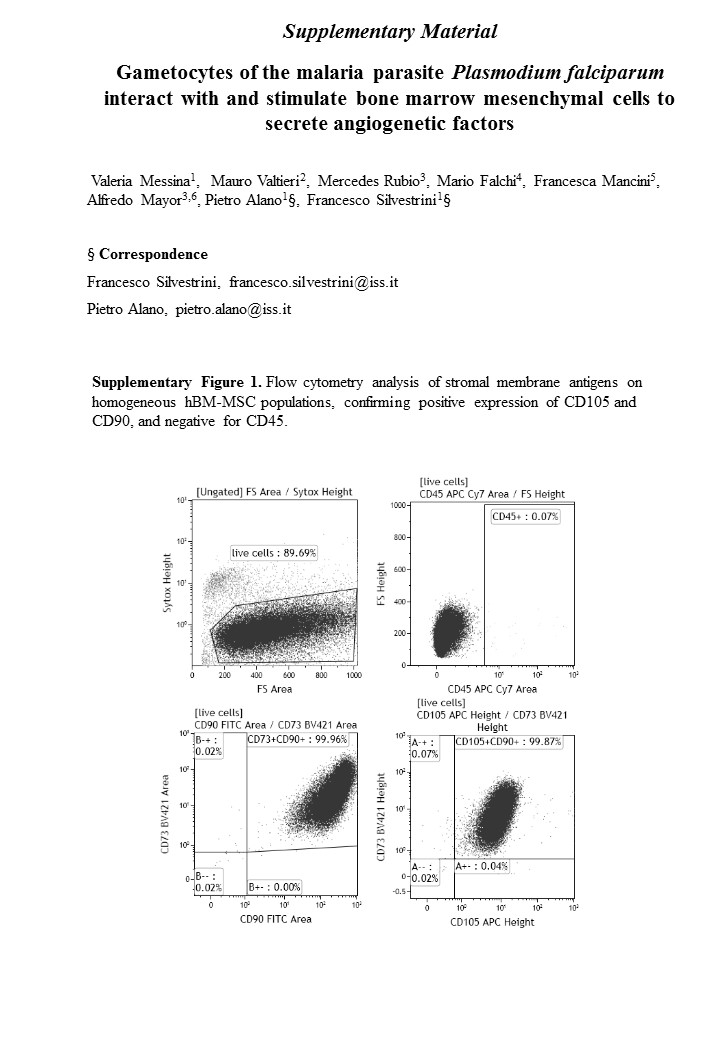

Supplement: Supplementary file 1 [file Image1.JPEG]

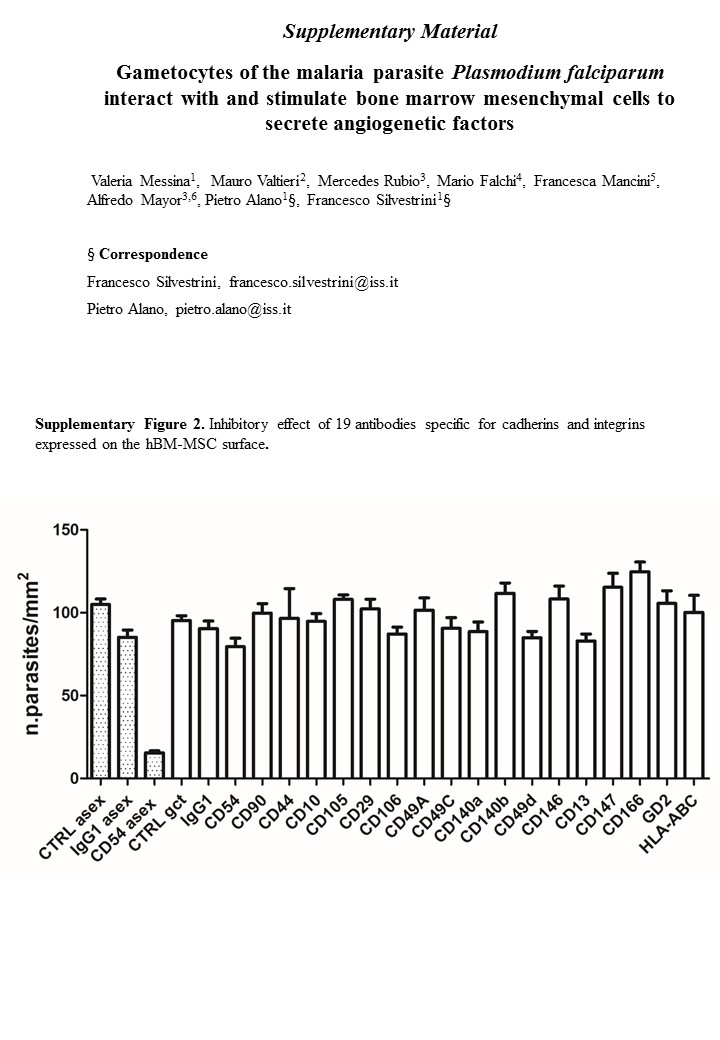

Supplement: Supplementary file 2 [file Image2.JPEG]

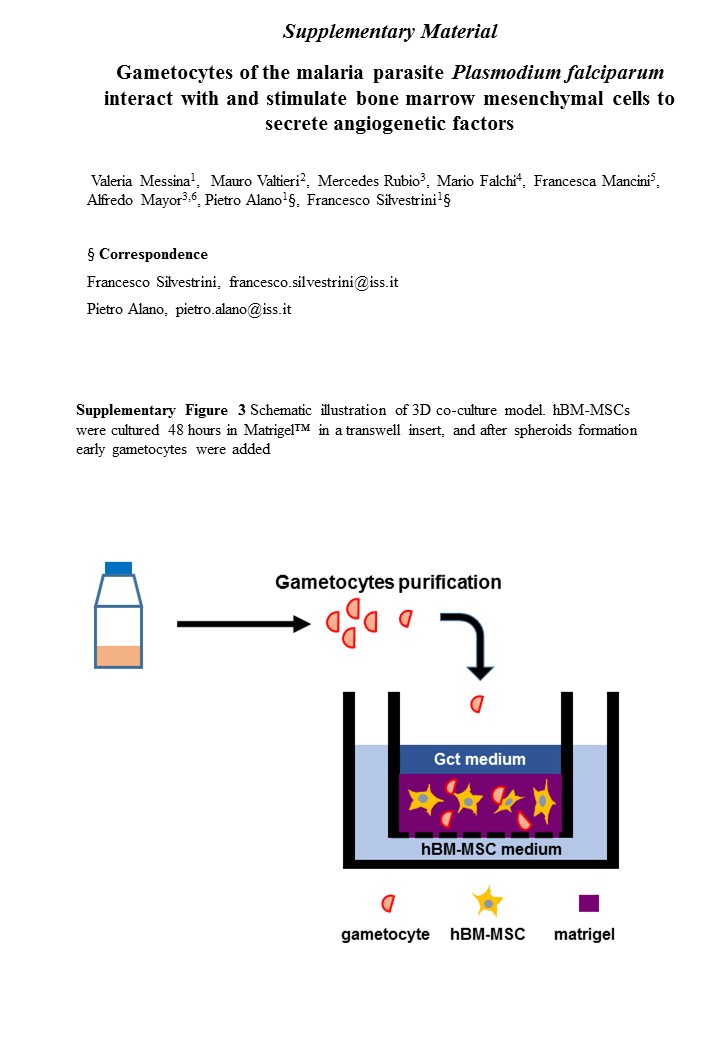

Supplement: Supplementary file 3 [file Image3.JPEG]

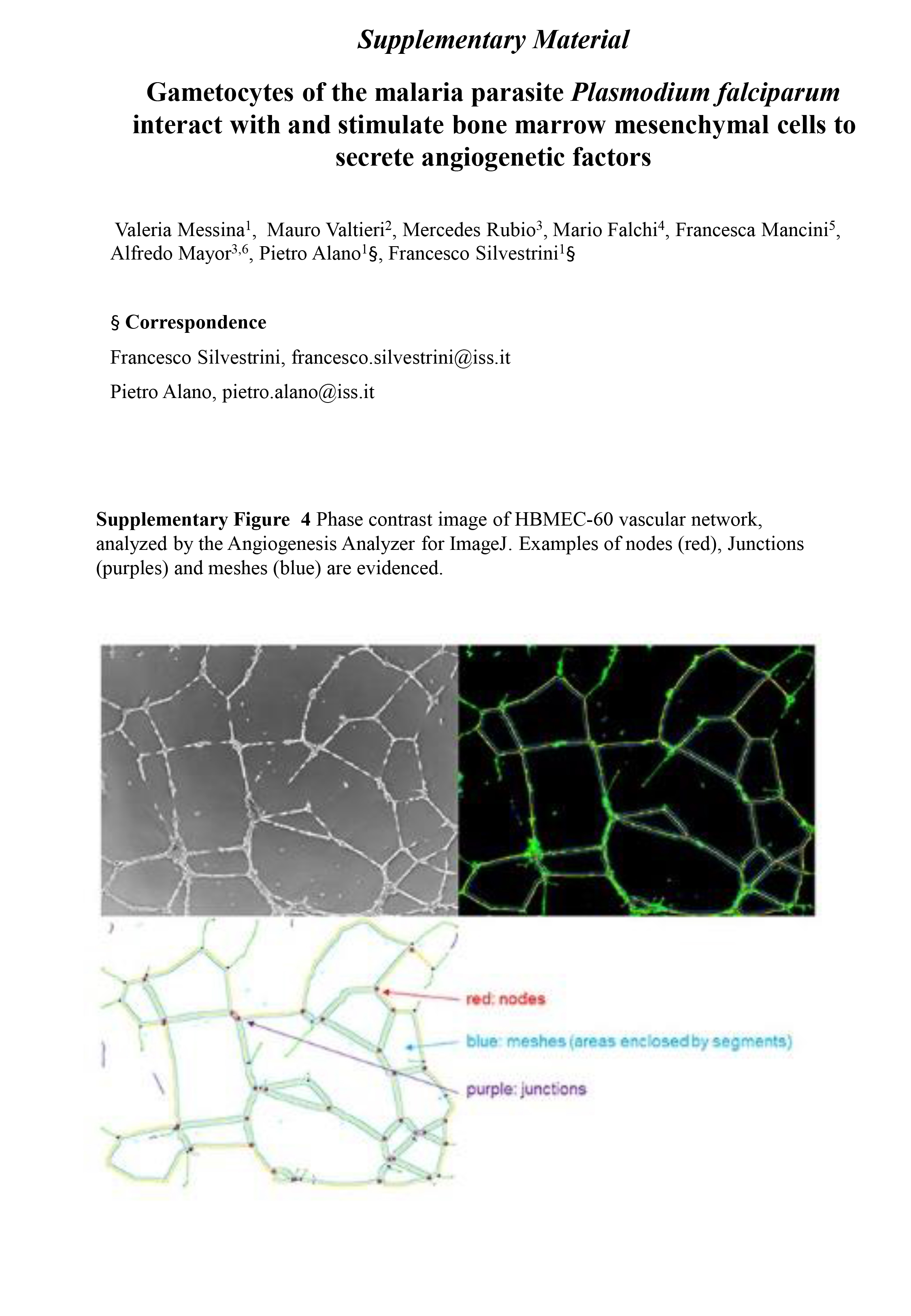

Supplement: Supplementary file 4 [file Image4.jpg]
