# Supplementary material for: Gametocytes of the Malaria Parasite Plasmodium falciparum Interact With and Stimulate Bone Marrow Mesenchymal Cells to Secrete Angiogenetic Factors
Source: Front Cell Infect Microbiol. 2018 Mar 1;8:50. doi: 10.3389/fcimb.2018.00050 (PMC5838020; doi:10.3389/fcimb.2018.00050)
Supplement: Supplementary file 5 [file Table1.PDF]

# *Supplementary Material*

## Gametocytes of the malaria parasite *Plasmodium falciparum* interact with and stimulate bone marrow mesenchymal cells to secrete angiogenetic factors

### Supplementary Table 1

| pg/mL/1000 cells | IL8 * | | | | IL6 * | | | | CCL2 * | | | | CXCL9 *** | | | | VEGF ** | | | | IL1b ** | | | |
|---|---|---|---|---|---|---|---|---|---|---|---|---|---|---|---|---|---|---|---|---|---|---|---|---|
| | hMSC | | hMSC+GCT | | hMSC | | hMSC+GCT | | hMSC | | hMSC+GCT | | hMSC | | hMSC+GCT | | hMSC | | hMSC+GCT | | hMSC | | hMSC+GCT | |
| | rep1 | rep2 | rep1 | rep2 | rep1 | rep2 | rep1 | rep2 | rep1 | rep2 | rep1 | rep2 | rep1 | rep2 | rep1 | rep2 | rep1 | rep2 | rep1 | rep2 | rep1 | rep2 | rep1 | rep2 |
| day2 | 1.2853 | 1.2901 | 4.4740 | 2.5017 | 1.8844 | 0.2821 | 0.8488 | 2.0825 | 4.2710 | 3.8928 | 11.3895 | 9.1044 | 0.0384 | 0.0392 | 0.5733 | 0.6266 | 2.1116 | 6.5546 | 9.5353 | 10.2058 | 0.1087 | 0.3812 | 0.9232 | 0.6684 |
| day4 | 0.9837 | 2.5405 | 67.3798 | 162.2073 | 0.5930 | 0.2875 | 10.6714 | 14.1899 | 1.3155 | 5.3452 | 16.3306 | 27.2835 | 0.0371 | 0.0399 | 0.7794 | 0.5086 | 0.0894 | 0.0752 | 12.4513 | 14.8909 | 0.1948 | 0.6427 | 0.5026 | 0.9757 |
| day6 | 1.5353 | 1.9146 | 132.6037 | 178.0588 | 0.1792 | 0.1122 | 7.1709 | 11.9262 | 5.5833 | 2.6299 | 27.4909 | 35.4283 | 0.0358 | 0.0301 | 0.4218 | 0.6394 | 0.0926 | 0.0998 | 14.5249 | 19.9366 | 0.5762 | 0.4844 | 0.6793 | 1.1916 |
| day8 | 1.5014 | 4.8113 | 135.8716 | 205.4369 | 0.1842 | 0.3286 | 20.3098 | 32.6478 | 6.8717 | 4.7785 | 37.7102 | 48.3584 | 0.0346 | 0.0329 | 0.4941 | 0.6811 | 0.0865 | 0.8145 | 16.5134 | 23.8994 | 0.6635 | 0.5305 | 0.7957 | 1.0019 |

| | HGF * | | | | GM-CSF ns | | | | FGF ** | | | | CCL3 ** | | | | IL12 ns | | | | INFg ns | | | |
|---|---|---|---|---|---|---|---|---|---|---|---|---|---|---|---|---|---|---|---|---|---|---|---|---|
| | hMSC | | hMSC+GCT | | hMSC | | hMSC+GCT | | hMSC | | hMSC+GCT | | hMSC | | hMSC+GCT | | hMSC | | hMSC+GCT | | hMSC | | hMSC+GCT | |
| | rep1 | rep2 | rep1 | rep2 | rep1 | rep2 | rep1 | rep2 | rep1 | rep2 | rep1 | rep2 | rep1 | rep2 | rep1 | rep2 | rep1 | rep2 | rep1 | rep2 | rep1 | rep2 | rep1 | rep2 |
| day2 | 1.7627 | 0.4701 | 6.1709 | 3.2098 | 0.0141 | 0.0144 | 0.0211 | 0.0153 | 11.2075 | 14.0132 | 22.0335 | 15.3276 | 1.8537 | 1.8887 | 5.0905 | 3.5597 | 0.3457 | 0.3523 | 1.9159 | 1.0612 | 0.9608 | 1.0150 | 1.8699 | 1.3000 |
| day4 | 1.6997 | 1.8305 | 0.6197 | 8.4509 | 0.0136 | 0.0147 | 0.0190 | 0.0519 | 9.1096 | 11.9695 | 15.9476 | 25.6517 | 1.4847 | 2.5144 | 2.4901 | 4.6685 | 0.3334 | 0.3590 | 0.9022 | 1.5015 | 0.5142 | 1.0951 | 0.4846 | 1.7250 |
| day6 | 3.8515 | 3.2375 | 5.7863 | 7.0366 | 0.0132 | 0.0111 | 0.1051 | 0.0658 | 6.9978 | 10.8785 | 18.7143 | 15.9999 | 2.7328 | 2.2971 | 3.4878 | 3.5020 | 1.1958 | 0.5256 | 0.7372 | 1.2502 | 1.0744 | 1.1370 | 1.3758 | 1.6010 |
| day8 | 3.7231 | 4.5188 | 6.7783 | 10.0508 | 0.0127 | 0.0121 | 0.1017 | 0.1562 | 4.9410 | 9.5725 | 19.3986 | 14.1179 | 2.6417 | 2.5159 | 3.9311 | 5.0371 | 1.1559 | 0.7102 | 1.6513 | 1.5077 | 1.2178 | 1.5598 | 1.5679 | 1.9397 |

| | TNFa ns | | | | IL2 ns | | | | IL7 * | | | | CXCL10 * | | | | IL2R ns | | | | IL4 ns | | | |
|---|---|---|---|---|---|---|---|---|---|---|---|---|---|---|---|---|---|---|---|---|---|---|---|---|
| | hMSC | | hMSC+GCT | | hMSC | | hMSC+GCT | | hMSC | | hMSC+GCT | | hMSC | | hMSC+GCT | | hMSC | | hMSC+GCT | | hMSC | | hMSC+GCT | |
| | rep1 | rep2 | rep1 | rep2 | rep1 | rep2 | rep1 | rep2 | rep1 | rep2 | rep1 | rep2 | rep1 | rep2 | rep1 | rep2 | rep1 | rep2 | rep1 | rep2 | rep1 | rep2 | rep1 | rep2 |
| day2 | 0.1169 | 0.1191 | 0.4133 | 0.1262 | 0.0687 | 0.0279 | 0.3392 | 0.1597 | 4.2338 | 0.0285 | 6.3156 | 4.7169 | 0.1143 | 0.1164 | 0.4289 | 0.2140 | 11.3101 | 6.0792 | 24.4090 | 11.0920 | 0.0712 | 0.0726 | 0.4287 | 0.0769 |
| day4 | 0.1127 | 0.2312 | 0.0890 | 0.1547 | 0.0264 | 0.0713 | 0.1454 | 0.1432 | 4.2115 | 3.7512 | 7.0751 | 6.6387 | 0.1102 | 0.1792 | 0.2318 | 0.2940 | 8.8891 | 8.4655 | 8.0148 | 11.4976 | 0.0687 | 0.0739 | 0.0957 | 0.2036 |
| day6 | 0.1575 | 0.1324 | 0.2444 | 0.1864 | 0.1008 | 0.0847 | 0.1188 | 0.1848 | 3.8790 | 0.0219 | 4.3483 | 4.2135 | 0.2677 | 0.1739 | 0.3267 | 0.3169 | 14.3091 | 9.6564 | 17.4149 | 12.4642 | 0.1432 | 0.0557 | 0.0782 | 0.1695 |
| day8 | 0.2004 | 0.1002 | 0.3211 | 0.2614 | 0.1688 | 0.1267 | 0.2412 | 0.2202 | 3.5656 | 0.0240 | 4.8258 | 6.4730 | 0.2945 | 0.2003 | 0.3956 | 0.6564 | 15.6152 | 9.6944 | 22.3074 | 13.8824 | 0.2586 | 0.0610 | 0.1977 | 0.0836 |

| | INFa ns | | | | IL15 ns | | | | CCL11 ns | | | | G-CSF * | | | | IL10 * | | | | IL13 ns | | | |
|---|---|---|---|---|---|---|---|---|---|---|---|---|---|---|---|---|---|---|---|---|---|---|---|---|
| | hMSC | | hMSC+GCT | | hMSC | | hMSC+GCT | | hMSC | | hMSC+GCT | | hMSC | | hMSC+GCT | | hMSC | | hMSC+GCT | | hMSC | | hMSC+GCT | |
| | rep1 | rep2 | rep1 | rep2 | rep1 | rep2 | rep1 | rep2 | rep1 | rep2 | rep1 | rep2 | rep1 | rep2 | rep1 | rep2 | rep1 | rep2 | rep1 | rep2 | rep1 | rep2 | rep1 | rep2 |
| day2 | 0.0227 | 0.0232 | 2.4731 | 1.5595 | 0.1218 | 3.5070 | 5.1346 | 2.3139 | 0.0453 | 0.0353 | 0.2492 | 0.0804 | 5.1614 | 0.2450 | 7.6993 | 3.8717 | 0.2978 | 0.1802 | 0.5054 | 0.2341 | 0.4990 | 0.8609 | 1.1347 | 0.5389 |
| day4 | 0.0219 | 0.0236 | 0.0306 | 2.1943 | 0.1174 | 4.7861 | 0.1636 | 7.5364 | 0.0268 | 0.1448 | 0.0133 | 0.1691 | 3.4569 | 0.2497 | 0.3230 | 10.5469 | 0.2090 | 0.2251 | 0.4552 | 0.2869 | 0.7335 | 0.7010 | 0.9068 | 1.1183 |
| day6 | 0.8758 | 0.9458 | 1.8196 | 1.8271 | 3.2047 | 5.2607 | 2.3515 | 2.3611 | 0.1929 | 0.1527 | 0.1617 | 0.1408 | 4.8055 | 4.0394 | 5.6650 | 8.7819 | 0.2018 | 0.1696 | 0.2821 | 0.2389 | 0.5474 | 0.4601 | 0.5477 | 0.8383 |
| day8 | 1.8380 | 0.0195 | 2.1316 | 2.3974 | 5.1248 | 2.9503 | 4.4255 | 6.6845 | 0.1792 | 0.1741 | 0.2768 | 0.1591 | 4.6453 | 4.4241 | 6.6361 | 9.3546 | 0.2680 | 0.1180 | 0.2274 | 0.3496 | 1.6846 | 0.6520 | 0.9232 | 1.0894 |

| | CCL5 ns | | | | IL17 ns | | | | CCL4 ns | | | | IL5 ns | | | | IL1RA ns | | | | EGF ** | | | |
|---|---|---|---|---|---|---|---|---|---|---|---|---|---|---|---|---|---|---|---|---|---|---|---|---|
| | hMSC | | hMSC+GCT | | hMSC | | hMSC+GCT | | hMSC | | hMSC+GCT | | hMSC | | hMSC+GCT | | hMSC | | hMSC+GCT | | hMSC | | hMSC+GCT | |
| | rep1 | rep2 | rep1 | rep2 | rep1 | rep2 | rep1 | rep2 | rep1 | rep2 | rep1 | rep2 | rep1 | rep2 | rep1 | rep2 | rep1 | rep2 | rep1 | rep2 | rep1 | rep2 | rep1 | rep2 |
| day2 | 17.7248 | 19.5012 | 56.1862 | 41.7071 | 1.8844 | 0.2821 | 0.8488 | 2.0825 | 1.0232 | 0.5081 | 1.5501 | 0.8328 | 0.0235 | 0.0239 | 0.0350 | 0.0253 | 0.1135 | 0.1156 | 0.1692 | 0.1225 | 0.7249 | 0.4814 | 2.5548 | 1.3970 |
| day4 | 11.6634 | 33.5030 | 16.5722 | 49.5666 | 0.5930 | 0.2875 | 0.6714 | 14.1899 | 1.4809 | 0.8008 | 0.6699 | 2.4179 | 0.0226 | 0.0244 | 0.0315 | 0.2220 | 0.1094 | 0.1097 | 0.1524 | 0.1502 | 0.4556 | 1.1149 | 0.6347 | 2.4010 |
| day6 | 34.0335 | 30.3492 | 39.6887 | 46.3949 | 0.1792 | 0.1122 | 0.1709 | 11.9262 | 0.7179 | 1.0205 | 1.4312 | 1.7263 | 0.1562 | 0.1313 | 0.0257 | 0.1848 | 0.1056 | 0.0888 | 0.1245 | 3.5774 | 1.2043 | 1.2600 | 1.6530 | 2.7514 |
| day8 | 36.2163 | 57.2030 | 56.7938 | 56.5085 | 0.1842 | 0.3286 | 0.3098 | 32.6478 | 0.9352 | 2.8907 | 2.3488 | 2.4483 | 0.1510 | 0.0201 | 0.0302 | 0.0275 | 0.1021 | 0.0972 | 0.1459 | 0.1332 | 1.4024 | 1.2005 | 2.0700 | 3.3687 |

* p<0.05
** p<0.01
*** p<0.001
ns: not significant
